# Supplementary material for: Metallic nanocrystals with low angle grain boundary for controllable plastic reversibility
Source: Nat Commun. 2020 Jun 18;11:3100. doi: 10.1038/s41467-020-16869-3 (PMC7303210; doi:10.1038/s41467-020-16869-3)
Supplement: Supplementary file 1 — Supplementary Information [file 41467_2020_16869_MOESM1_ESM.pdf]

Metallic nanocrystals with low angle grain boundary for controllable plastic reversibility

Zhu et al.

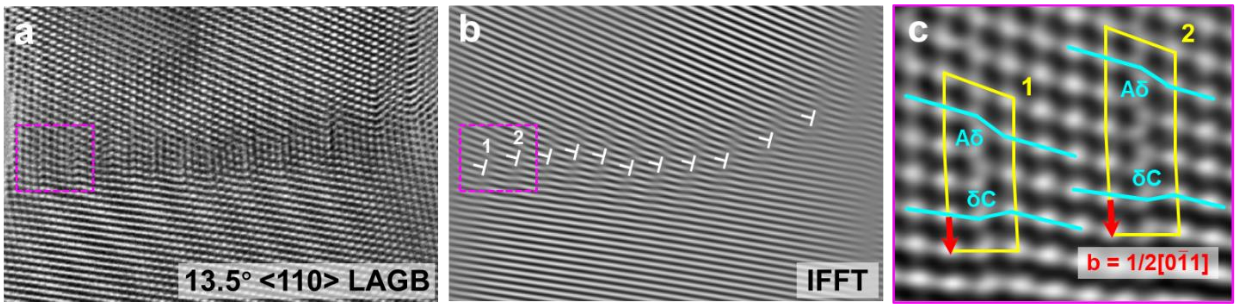

Supplementary Figure 1. (a-b) Original HRTEM image and inverse fast Fourier transform (IFFT) image showing the dislocations at the 13.5° <110> LAGB. (c) Atomistic structure of the Shockley partial pairs (**δC** and **Aδ**) that constitute dislocations No. 1 and 2 at the LAGB.

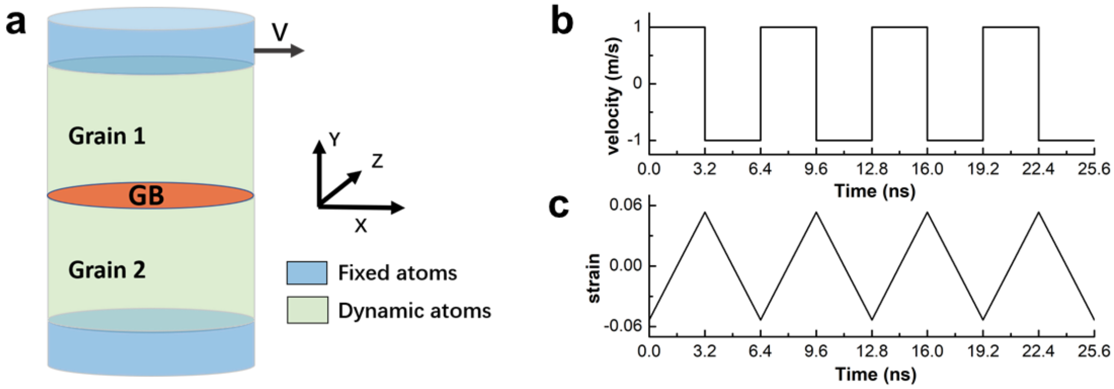

Supplementary Figure 2. (a) Schematic showing the set-up of the cylindrical bicrystal simulation cell used in this work. A shear velocity was applied to the rigid slab (consisting of fixed atoms) of the top grain. (b) Reversible shear velocity with an amplitude of 1 m s^{-1} applied in loading cycles. (c) Shear strain profile in loading cycles.

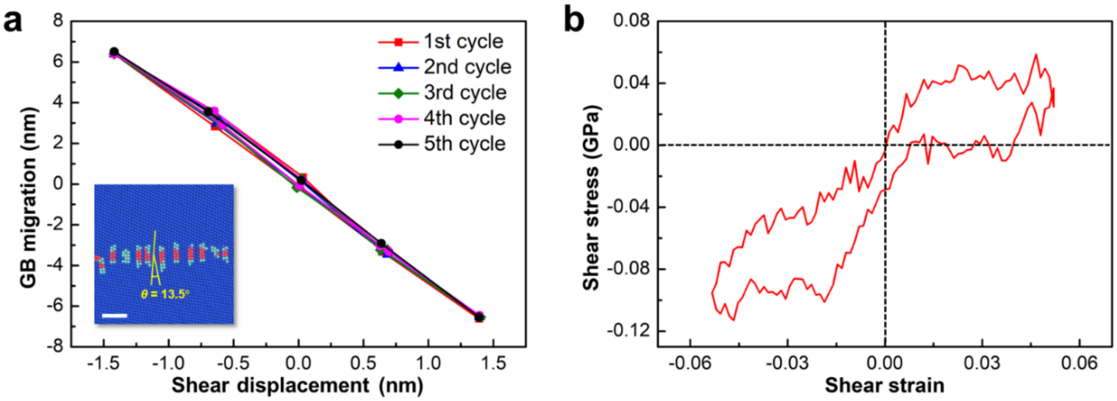

Supplementary Figure 3. (a) Grain boundary (GB) migration-shear displacement curves of a 13.5° <110> low angle GB (LAGB, see inset) during the reversible deformation of an Au bicrystal in molecular dynamics (MD) simulation. (b) The stress-strain curve corresponding to the first shear loading cycle of the Au bicrystal. Scale bar: 2 nm.

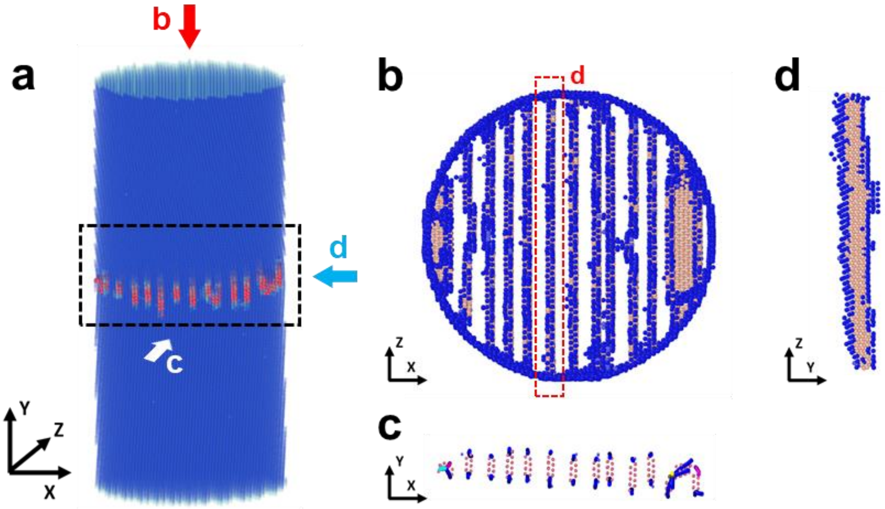

Supplementary Figure 4. Atomistic characterizations of dissociated GB dislocations in MD simulations from different viewing directions. (a) Perspective view of orderly-aligned dissociated GB dislocations (marked out by the dashed rectangle) inside an Au bicrystal. (b) Plain view of the GB boundary slab consisting of uniformly distributed partial dislocations pairs. (c) Front view of orderly-aligned Shockley partial dislocation pairs bound with stacking fault ribbons (red atoms). (d) Side view of an individual pair of straight Shockley partial dislocations marked out in (b).

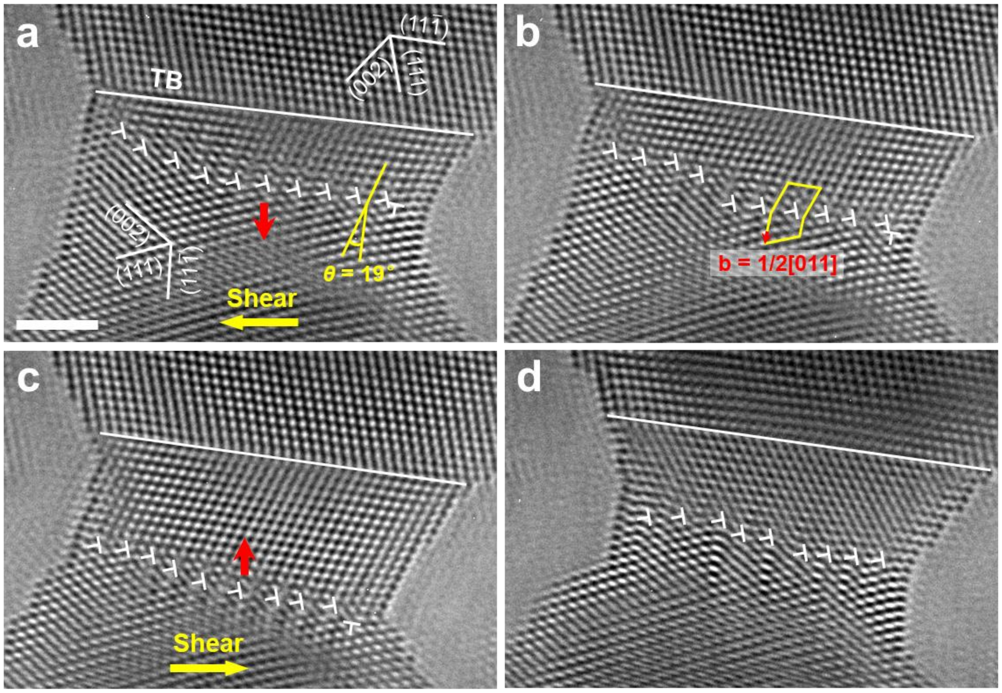

Supplementary Figure 5. Reversible migration of a 19° [1$\bar{1}$0] LAGB in a shear loading cycle via the dissociated GB dislocations. Scale bar: 2 nm.

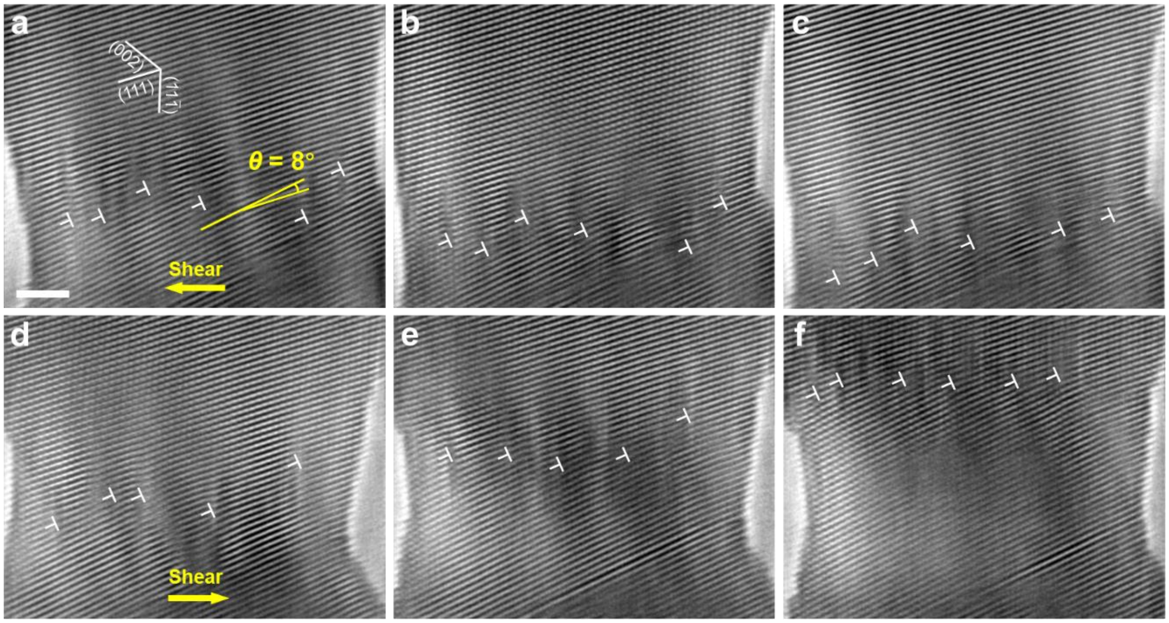

Supplementary Figure 6. Reversible migration of an 8° [1$\bar{1}$0] LAGB in a shear loading cycle via the dissociated GB dislocations. Scale bar: 2 nm.

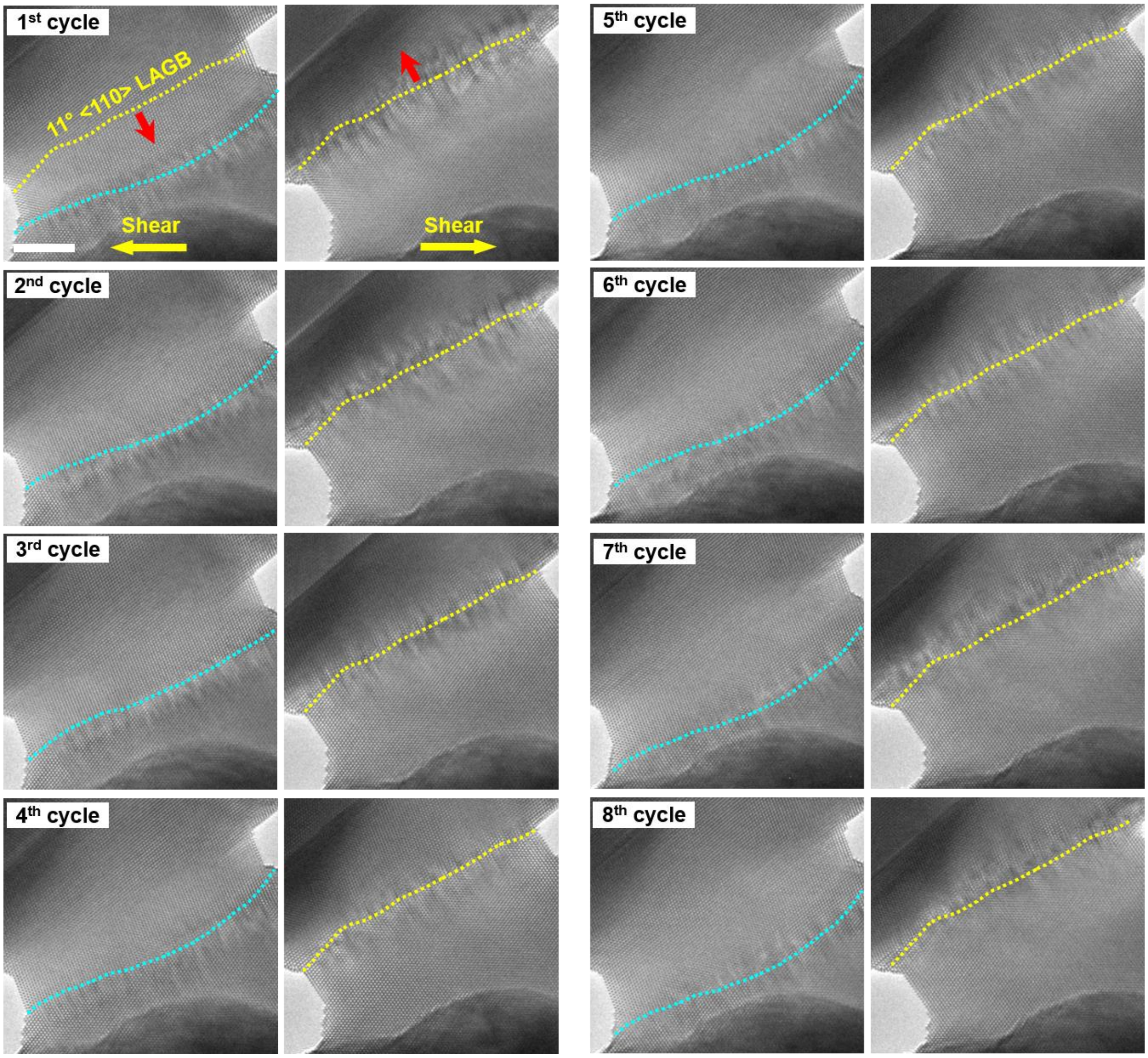

Supplementary Figure 7. Well-retained reversible migration of 11° [1$\bar{1}$0] LAGB in eight shear loading cycles. The yellow and light blue dotted lines delineate the top and bottom positions of the GB. Scale bar: 5 nm.

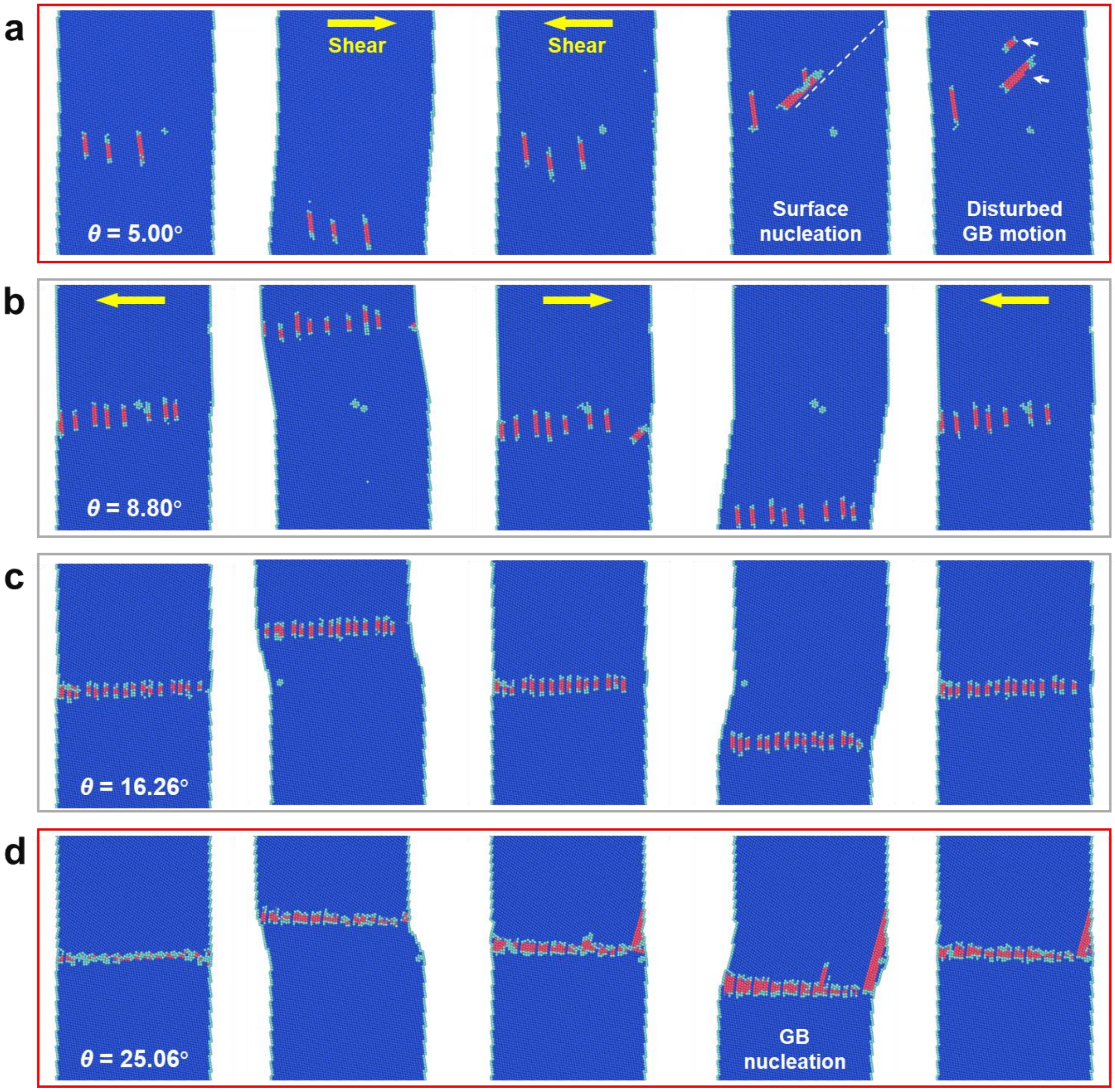

Supplementary Figure 8. MD simulations showing different reversible deformation behavoiurs of various <110> tilt GBs in Au bicrystals. (a) $\theta = 5.00°$, (b) $\theta = 8.80°$, (c) $\theta = 16.26°$ and (d) $\theta = 25.06°$. The cyclic deformability of 5.00 <110> GB was compromised, due to the interaction with additional lattice defects that nucleated from the free surface. In contrast, the cyclic deformability was generally retained in all other three characteristic GBs with larger misorientations. Nevertheless, defect nucleation from the GB was occasionally observed in the bicrystal with a 25.06° <110> GB, indicating the upper limit of misorientation for the GB-dominated reversible deformation.

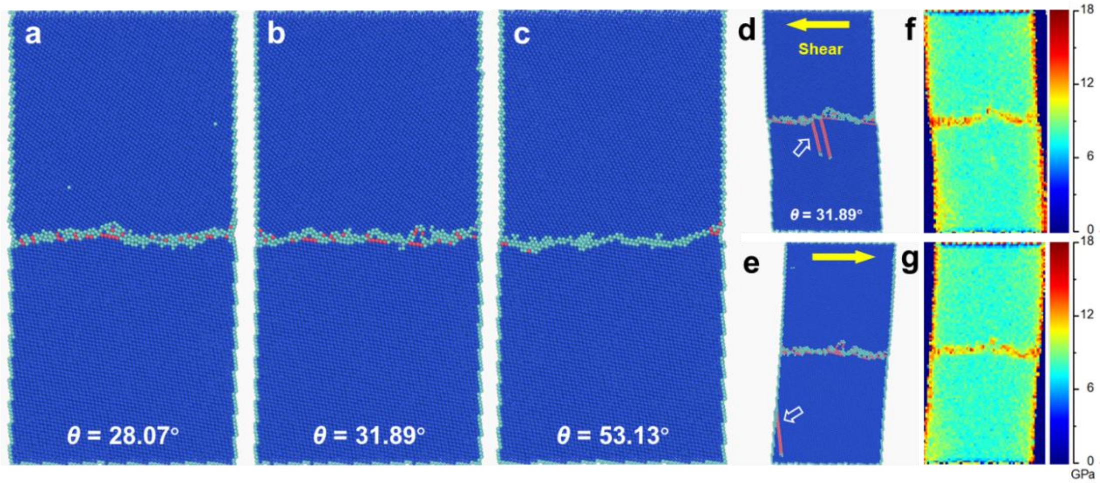

Supplementary Figure 9. Failure of GB-mediated reversible deformations in Au bicrystals with larger misorientations. (a-c) Disintegrated GB structures in Au bicrystals at 300 K with misorientations of (a) 28.07°, (b) 31.89° and (c) 53.13°, respectively. (d, e) Severely destabilized 31.89° <110> GB in a bicrystal under reversed shear loading. Non-conservative dislocation nucleation from either the GB or free surface are indicated by the white arrows. (f, g) Stress field maps of deformed bicrystals in (d, e) demonstrating severe stress concentration at the GB.

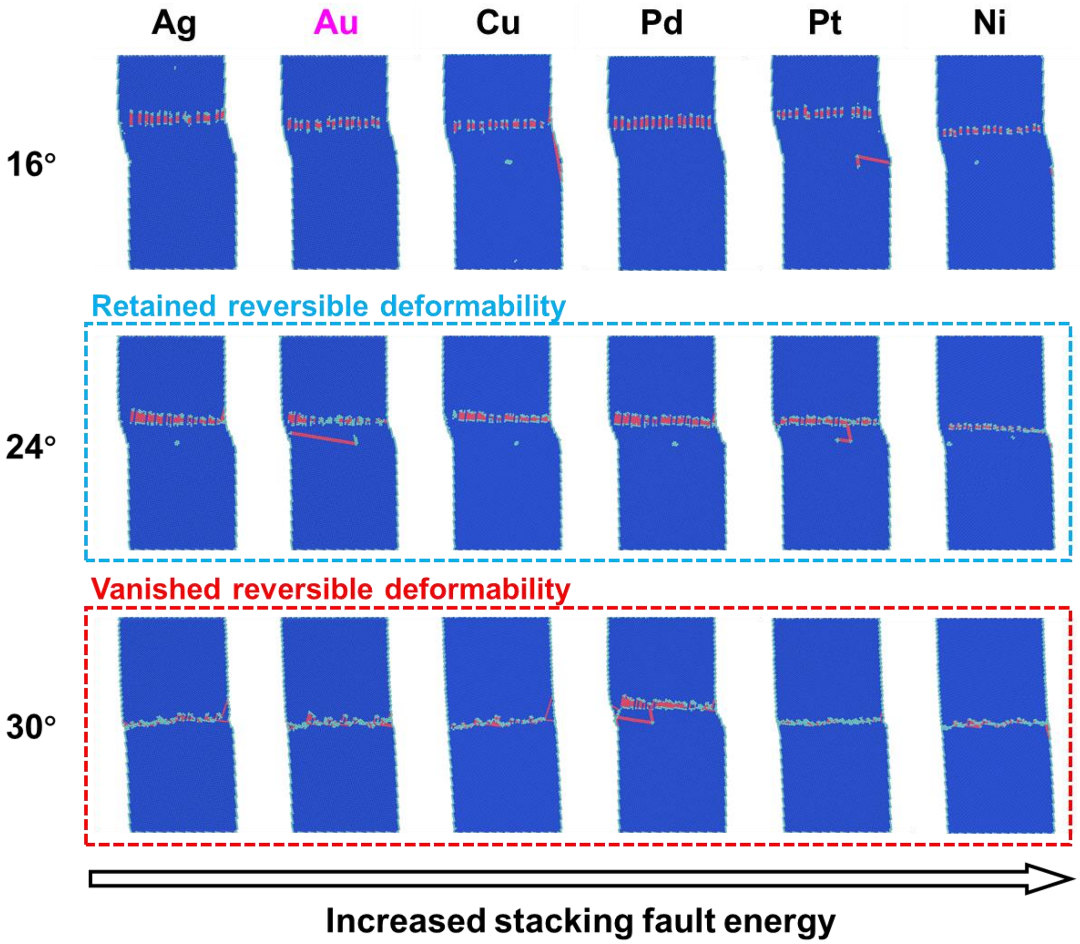

Supplementary Figure 10. Reversible deformability with respect to GB misorientation in different face-centred cubic (FCC) bicrystals. At θ = 16°, GB dissociation prevail among different metals, where the reversible deformability is well-retained. The different dissociation widths are closely related to the stacking fault energies (SFEs) of each FCC metal. At θ = 24°, the reversible deformability of the GBs is generally retained, while the GB dissociation widths are reduced. Notably, the GB in Ni bicrystal seems to have reached a critical misorientation with limited dissociation. At θ = 30°, the stably dissociated GB configurations disappear in all metallic bicrystals (except for Pd), and the reversible deformability almost vanishes among all the FCC bicrystals. Instead, non-conservative nucleation of lattice defects from either the surface or GB prevail throughout the deformation.

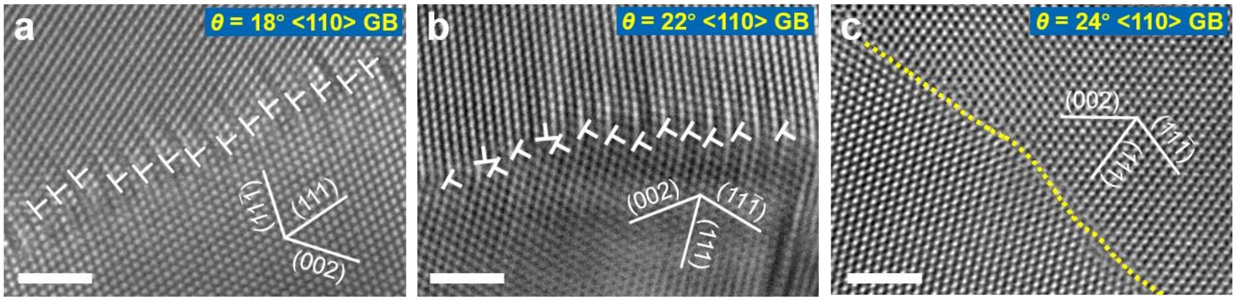

Supplementary Figure 11. Atomistic structures of the <110> tilt GBs with different misorientations. (a) θ = 18°, (b) θ = 22° and (c) θ = 24°. Evident contrasts of dissociations are shown in the 18° and 22° <110> GBs, while no observable dissociation occurs in the 24° <110> GB. Scale bars: 2 nm.

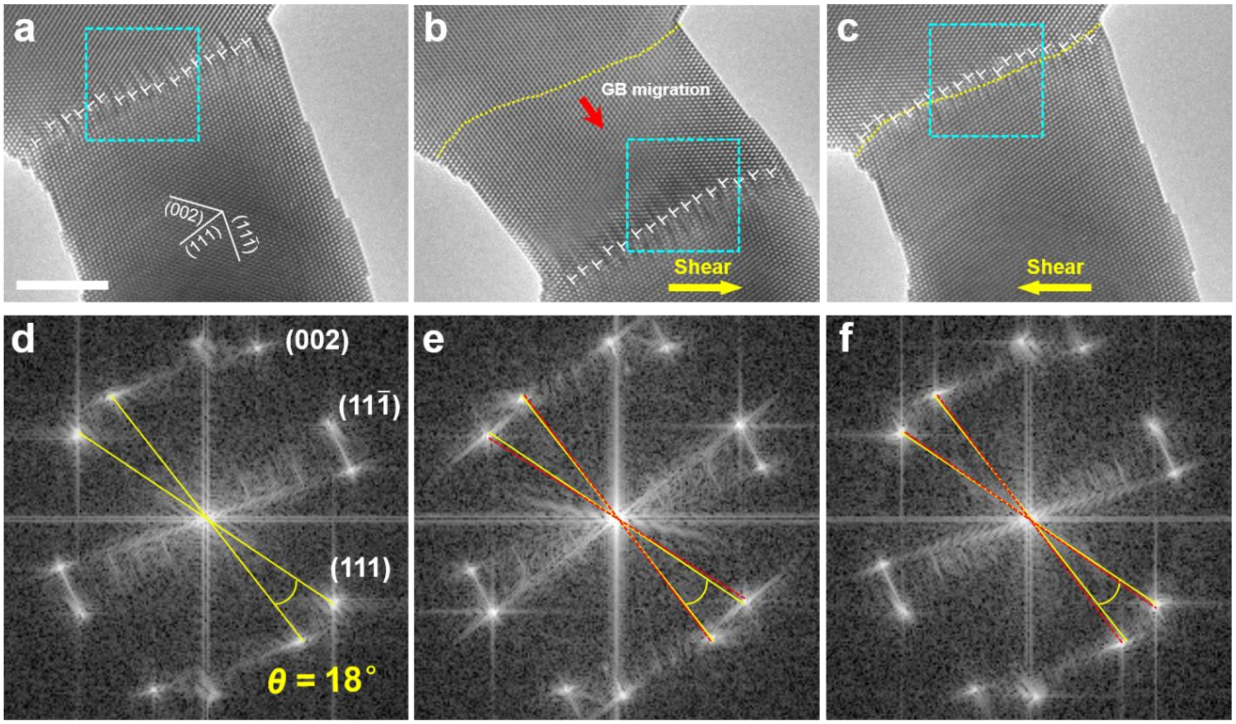

Supplementary Figure 12. Atomistic structures and lattice orientations of an Au bicrystal consisting of the 18° <110> GB during reversible shear loading. (a-c) Atomistic structure of a 18° <110> GB prior to shear loading (a), migrating to the bottom (b) and returning to its initial position (c). (d-f) Fast Fourier transform (FFT) patterns corresponding to the GB structures marked out by the light blue squares in (a-c). The superimposed red dashed lines and yellow solid lines mark the initial and real-time orientations of the top/bottom grain lattices, which remain overlapped throughout the reversible migration process, indicating the absence of lattice reorientation induced by bending or grain rotation. Scale bar: 5 nm.

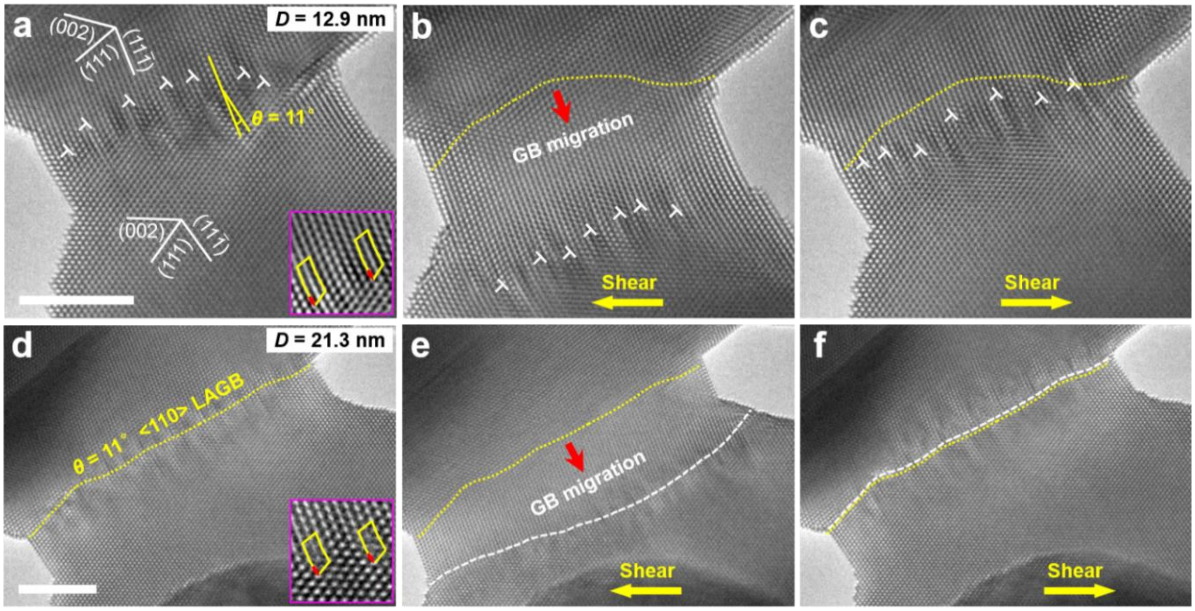

Supplementary Figure 13. Reversible migration of a 11° <110> LAGB in two Au bicrystals with different diameters. (a-c) *D* = 12.9 nm and (d-f) *D* = 21.3 nm. The insets in (a) and (d) show the atomistic structures of the dissociated GBs. The initial positions of the GBs are shown by the yellow dotted lines. Scale bars: 5 nm.

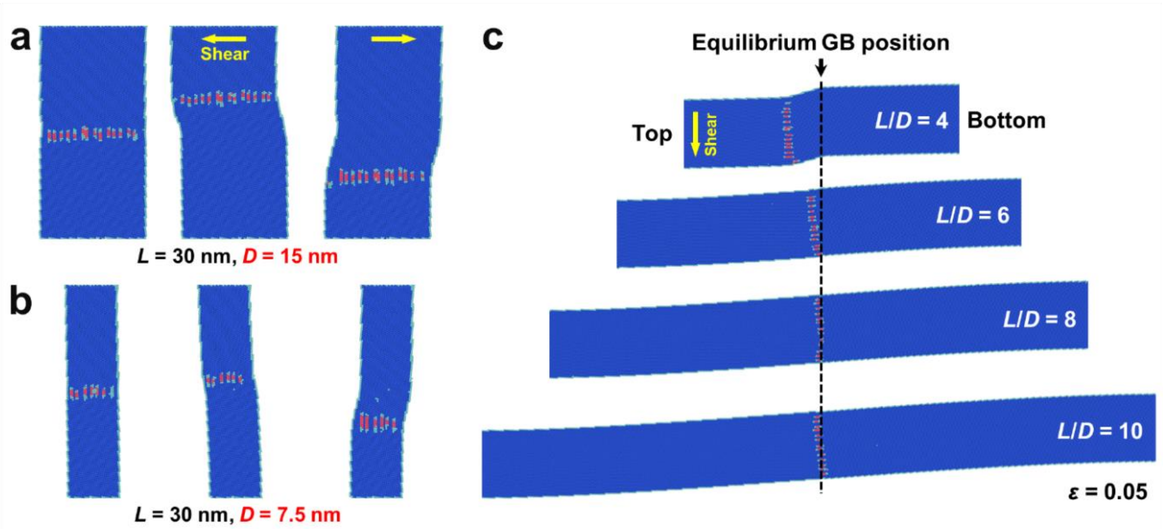

Supplementary Figure 14. Effects of diameter and aspect ratio on the reversible deformation of Au bicrystals with a 13.5° $[1\bar{1}0]$ LAGB. (a-b) GB-mediated reversible deformation of bicrystals with different diameters. The reversible deformability is well-retained in the bicrystal with half the diameter. (c) GB migration in four bicrystals with different aspect ratios (*L*/*D*) under the shear strain of 0.05. The bicrystals are rotated 90° for clear comparison of the GB migration distances and the black dashed line indicates the original positions of the GBs in each bicrystal. It is unambiguously shown that negligible GB motion is activated when *L*/*D* > 6.

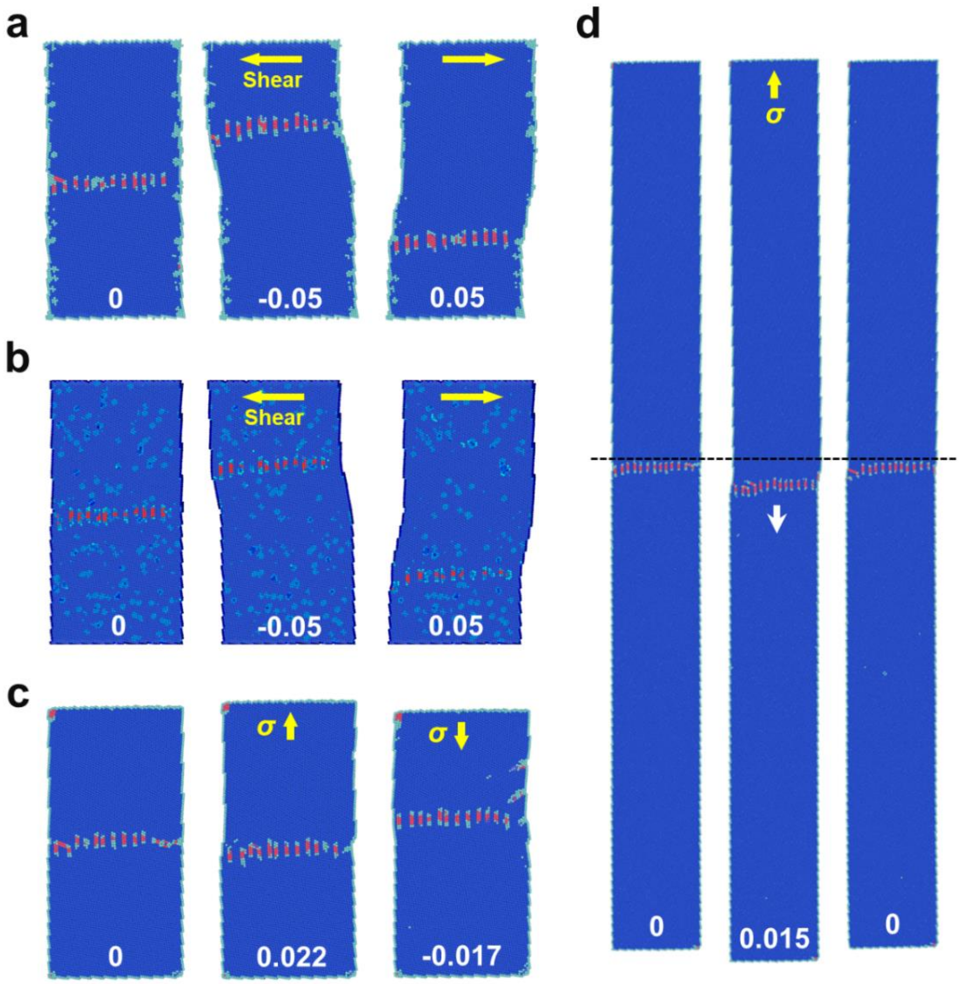

Supplementary Figure 15. (a) Reversible GB migration in an Au bicrystal with high level of surface roughness in shear loading cycle. The rough surface was constructed by randomly removing 20% of the surface atoms. (b) Reversible GB migration in an Au bicrystal with a vacancy density of 1% in shear loading cycle. (c) GB migration mediated reversible deformation of an Au bicrystal (*L*/*D*=2) in uniaxial tension/compression loading cycle. (d) GB migration mediated reversible deformation of an Au bicrystal nanowire (*L*/*D*=10) under uniaxial tension-unloading cycle. The black dashed line marks the initial position of the LAGB to clearly show the migration distance of the LAGB. The GBs in above bicrystal nanowires are 13.5° <110> LAGBs, and the nominal strains are clearly indicated in each snapshot.

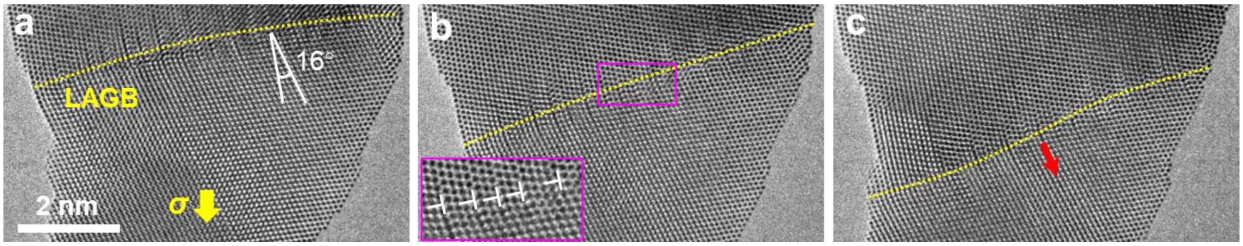

Supplementary Figure 16. Migration a 16° <110> LAGB governed by the conservative motion of GB dislocations under uniaxial tensile loading

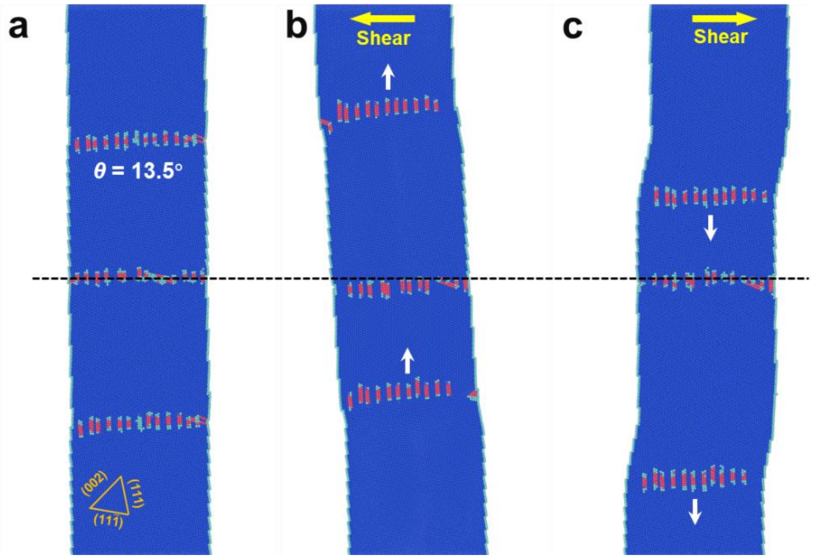

Supplementary Figure 17. Reversible migration of parallel GBs under cyclic shear loading. (a) As-received Au nanowire with three parallel 13.5° [1$\bar{1}$0] LAGBs. (b-c) Reversible migration (shown by the white arrows) of both top and bottom LAGBs in a shear loading cycle. This cylindrical nanocrystal possesses a diameter of 15 nm and a length of 45 nm.

Supplementary Table 1. Parameters of all bicrystal samples studied in experiments

<table>
<tr><th>No.</th><th>Misorientation</th><th>Ave. Diameter (nm)</th><th>Ave. Inclination</th><th>Strain rate</th><th>Reversible or not</th></tr>
<tr><td>1</td><td>7°</td><td>13.8</td><td>N/A</td><td rowspan="6">10^{-3} s^{-1}</td><td rowspan="12">YES</td></tr>
<tr><td>2</td><td rowspan="2">11°</td><td>12.9</td><td>28.5°</td></tr>
<tr><td>3</td><td>21.3</td><td>25.5°</td></tr>
<tr><td>4</td><td rowspan="2">13°</td><td>15.2</td><td>25.5°</td></tr>
<tr><td>5</td><td>10.5</td><td>20.0°</td></tr>
<tr><td>6</td><td>13.5°</td><td>16.2</td><td>7.5°</td></tr>
<tr><td>7</td><td rowspan="4">18°</td><td>15.8</td><td rowspan="3">25.3°</td><td>10^{-3} s^{-1}</td></tr>
<tr><td>8</td><td>15.8</td><td>10^{-2} s^{-1}</td></tr>
<tr><td>9</td><td>15.8</td><td>10^{-1} s^{-1}</td></tr>
<tr><td>10</td><td>8.9</td><td>29.6°</td><td rowspan="4">10^{-3} s^{-1}</td></tr>
<tr><td>11</td><td>19°</td><td>11.6</td><td>6.5°</td></tr>
<tr><td>12</td><td>22°</td><td>18.7</td><td>4.0°</td></tr>
<tr><td>13</td><td>24°</td><td>33.2</td><td>45.0°</td><td>NO</td></tr>
</table>

Supplementary Table 2. Parameters of all bicrystal samples studied in MD simulations

<table>
<tr><th>No.</th><th>Metal type</th><th>Misorientation</th><th>Length (nm)</th><th>Diameter (nm)</th><th>Shear velocity</th><th>Notes</th></tr>
<tr><td>1</td><td rowspan="24">Au</td><td>8.8°</td><td rowspan="13">30</td><td rowspan="17">15</td><td rowspan="18">1 m s^{-1}</td><td rowspan="9">GB-mediated reversible deformation (300 K)</td></tr>
<tr><td>2</td><td>10.39°</td></tr>
<tr><td>3</td><td>12.68°</td></tr>
<tr><td>4</td><td>13.5°</td></tr>
<tr><td>5</td><td>14.25°</td></tr>
<tr><td>6</td><td>16.26°</td></tr>
<tr><td>7</td><td>18.92°</td></tr>
<tr><td>8</td><td>22.62°</td></tr>
<tr><td>9</td><td>25.06°</td></tr>
<tr><td>10</td><td>28.07°</td><td rowspan="3">No cyclic deformability</td></tr>
<tr><td>11</td><td>31.89°</td></tr>
<tr><td>12</td><td>53.13°</td></tr>
<tr><td>13</td><td>13.5°</td><td>500 cycles</td></tr>
<tr><td>14</td><td rowspan="11">13.5°</td><td>60</td><td>L/D = 4</td></tr>
<tr><td>15</td><td>90</td><td>L/D = 6</td></tr>
<tr><td>16</td><td>120</td><td>L/D = 8</td></tr>
<tr><td>17</td><td>150</td><td>L/D = 10</td></tr>
<tr><td>18</td><td rowspan="12">30</td><td>7.5</td><td>L/D = 4</td></tr>
<tr><td>19</td><td rowspan="11">15</td><td>10 m s^{-1}</td><td rowspan="2">Stain rate effect</td></tr>
<tr><td>20</td><td>100 m s^{-1}</td></tr>
<tr><td>21</td><td rowspan="9">1 m s^{-1}</td><td>0 K</td></tr>
<tr><td>22</td><td>600 K</td></tr>
<tr><td>23</td><td>Rough surface</td></tr>
<tr><td>24</td><td>Vacancy (1%)</td></tr>
<tr><td>25</td><td>Ag</td><td rowspan="5">16°
24°
30°</td><td rowspan="5">Different stacking fault energies (SFEs)</td></tr>
<tr><td>26</td><td>Cu</td></tr>
<tr><td>27</td><td>Pd</td></tr>
<tr><td>28</td><td>Pt</td></tr>
<tr><td>29</td><td>Ni</td></tr>
<tr><td>30</td><td rowspan="2">Au</td><td rowspan="2">13.5°</td><td>30</td><td>15</td><td>0.1 m s^{-1}</td><td>Bicrystal T/C[a]</td></tr>
<tr><td>31</td><td>150</td><td>15</td><td>0.5 m s^{-1}</td><td>Nanowire T/R[b]</td></tr>
</table>

[a]T/C = tension/compression, [b]T/R = tension/relaxation

Supplementary Discussion

1. Quantifying the energy of dissociated GB dislocations.

The equilibrium GB energy was determined by calculating the energy increase of the lattices within 5 nm from the GB plane after 20 ps relaxation, which enabled us to obtain an average GB energy. As shown in Fig. 3b, the average GB energy rises steadily with increasing misorientation, which, however, did not reflect the localized energy state of the constituent GB dislocations. Here, we adopt another method to further quantify the average energy of the stacking fault ribbons bounded by Shockley partial dislocation pairs (defined as virtual stacking fault energy, v-SFE) in Au bicrystals.

Although the stacking fault ribbons bounded by the Shockley dislocation pairs at the GB are slightly curved, we still adopt a similar method as that for conventional planar stacking faults in bulk FCC metal, due to the negligible curvature. Specifically, we extract the v-SFE (γ_{vsf}) from MD simulations by calculating the average potential energy rise per atom in the dissociated region (i.e., shown by the red atom columns in Fig. 2d), following the equation below:

$$\gamma_{\mathrm{vsf}} = \frac{E_{\mathrm{fault}} - E_{\mathrm{fcc}}}{\rho_{\mathrm{atom}}} \tag{1}$$

The E_{fault} and E_{fcc} denote the average potential energy per atom from the GB dissociation region and conventional FCC lattice, where E_{fault} is highly orientation-dependent. ρ_{atom} represents the atomic planar density in the dissociated region (stacking fault ribbon), which can be approximated to that of close-packed (111) planes in conventional FCC metals. The projected area per atom on (111) plane can be derived as $A = \sqrt{3}a^2/4$, where a is the lattice constant of Au at room temperature (Supplementary Fig. 18a-b). Since four neighbouring planes were identified to be affected by the stacking fault[1] (Supplementary Fig. 18c-d), the v-SFE can be determined as follow:

$$\gamma_{\mathrm{vsf}} = \frac{16\sqrt{3}\left(E_{\mathrm{fault}} - E_{\mathrm{fcc}}\right)}{3a^2} \tag{2}$$

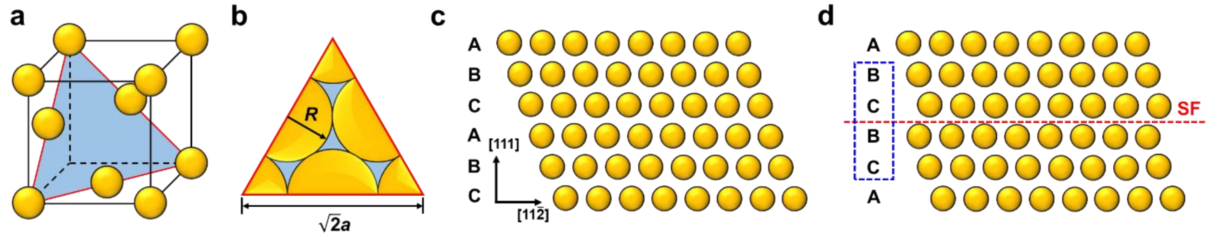

Supplementary Figure 18. (a) Perspective and (b) projective view of a (111) plane in FCC lattice. (c) Perfect FCC lattice viewed from [1$\bar{1}$0] direction. (d) FCC lattice with a SF (shown by a red dashed line). The blue column marks out the cut-off (111) atomic layers affected by the SF.

As shown in Supplementary Fig. 19, the v-SFE plot clearly exhibits three distinctive stages (unconfined, relaxed and restricted) in accordance with those of the GB dissociation widths, which can be ascribed to different configurations of GB dislocations and their interactions. Specifically, the v-SFE rises steadily first until ~12°, and then reaches a steady stage (12°~20°). Beyond 20°, the v-SFE elevates drastically until the upper limit of ~25°. Associated with this variation of v-SFE is the change of GB configurations: (i) θ < 12°, dissociated GB dislocation are distributed

discretely with negligible interaction; (ii) θ = 12°~20°, increased density of dissociated dislocations, while localized GB relaxation can still stabilize the v-SFE and benefit the dislocation dissociation; (iii) θ > 20°, large lattice misorientation induces an increasingly high level of stress concentration beyond the capability of localized relaxation. In the meantime, the interactions between GB dislocation cores start to prevail due to the oversaturation of dislocations at the GB[2], resulting in a drastically increased v-SFE and compromised GB stability.

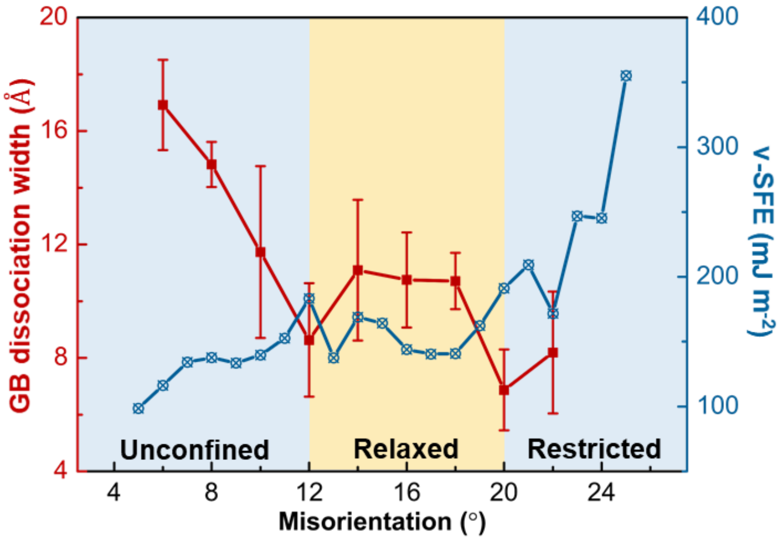

Supplementary Figure 19. Misorientation dependence of GB dissociation width and virtual stacking fault energy (v-SFE). Three near-identical misorientation stages are distinguished according to the trend of both GB dissociation width and v-SFE. These misorientation ranges correspond to the unconfined, relaxed and restricted configurations of the GB dislocations. Error bars represent the standard deviation from statistical analyses, where n ranges from 6 to 13, depending on misorientation.

2. Effect of aspect ratio on the reversible deformation of metallic bicrystals.

In a typical bicystal (L, D), the applied shear stress invariably induces a gradient bending moment $M = F_Q \cdot \lambda L$ on the nanocrystal, where F_Q (y) is the moment gradient along the axial direction of the nanocrystal (i.e., y-axis, see Supplementary Fig. 20a) and λL denotes the GB migration distance from its original position (in the middle). This gradient moment results in a normal stress distributed along the transverse direction (x-axis) of the nanocrystal $\sigma = M\mathrm{x}/I_z$ (Supplementary Fig. 20b), where I_z represents the moment of inertia ($I_z = \pi D^4/64$ for rods with a circular cross section). Therefore, the maximum normal stress on the GB plane induced by the moment can be derived as:

$$\sigma_{\max} = \frac{M \cdot D/2}{I_z} = \frac{F\lambda L/2 \cdot D/2}{\pi D^4/64} = 4\lambda\tau \cdot \frac{L}{D} \tag{3}$$

where τ is the shear stress applied on the top of the bicrystal. It is quite evident that the normal stress induced by the bending moment is proportional to the aspect ratio (L/D). According to the plane stress analysis (Supplementary Fig. 20c-d), this normal stress imposes a direct influence on the resolved shear stress on the (111) slip plane, which can be qualitatively expressed below:

$$\tau_n = \frac{\sigma_x - \sigma_y}{2}\sin 2\alpha + \tau_{xy}\cos 2\alpha = -\frac{\sigma}{2}\sin 2\alpha + \tau\cos 2\alpha \quad (4)$$

where α represents the angle between the (111) slip plane and the axial direction of the nanocrystal. A larger normal stress arising from the increasing aspect ratio (L/D) changes the original stress state on the slip plane, which could compromise the reversible migration of the GB. Therefore, the plastic reversibility in shear loading cycles is almost absent in nanocrystals with large aspect ratio.

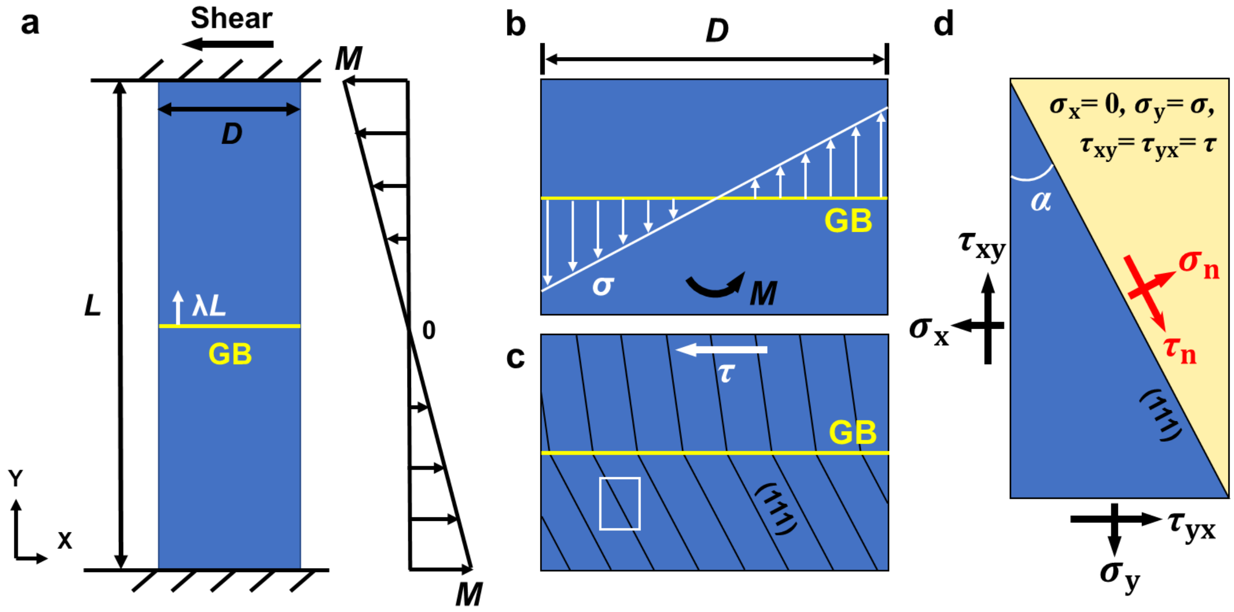

Supplementary Figure 20. Schematics demonstrating the aspect ratio effect on the GB-mediated deformation of a nanocrystal. (a) A gradient bending moment (*M*) induced by the applied shear stress on the nanocrystal. (b) Bending moment-induced gradient normal stress on the GB plane (indicated by the white arrows). (c) Schematic showing the coherent {111} slip planes in both top and bottom grains in (b). (d) Plane stress analysis of the equilibrium shear stress (τ_n) on the (111) slip plane, as marked out by the white rectangle in (c).

Supplementary References

1. Grujicic, M. & Zhou, X. W. Analysis of FeNiCrN austenite using the Embedded-Atom Method. *Calphad-computer Coupling of Phase Diagrams & Thermochemistry* **17**, 383-413 (1993).

2. Sutton, A. P. & Balluffi, R. W. *Interfaces In Crystalline Materials* (Oxford Univ. Press, Oxford, 1996).
