# Supplementary material for: Metallic nanocrystals with low angle grain boundary for controllable plastic reversibility
Source: Nat Commun. 2020 Jun 18;11:3100. doi: 10.1038/s41467-020-16869-3 (PMC7303210; doi:10.1038/s41467-020-16869-3)
Supplement: Supplementary file 3 — Description of Additional Supplementary Files [file 41467_2020_16869_MOESM3_ESM.pdf]

Description of Additional Supplementary Files

Supplementary Movie 1 Reversible deformation of an Au bicrystal containing a 13.5° [$1\bar{1}0$] LAGB. The GB migration was mediated by the collective motion of dissociated GB dislocations. The movie was speeded up by 5 times.

Supplementary Movie 2 Reversible migration of a 13.5° [$1\bar{1}0$] LAGB in Au bicrystal reproduced in MD simulation at 300 K under the shear velocity of 1 m s^{-1}.

Supplementary Movie 3 Real-time stress-strain curve corresponding to the reversible migration of the 13.5° [$1\bar{1}0$] LAGB in an Au bicrystal during shear loading cycle.

Supplementary Movie 4 Long-term reversible deformation of an Au bicrystal for 500 cycles in MD simulation.

Supplementary Movie 5 Reversible deformation of an Au bicrystal consisting a 18° [$1\bar{1}0$] GB under different strain rates. The movie was speeded up by 5 times for deformations under the strain rates of 0.006 s^{-1} and 0.06 s^{-1}.
